# Patient versus clinician-reported outcomes following tooth autotransplantation: part II of a retrospective cohort study

**DOI:** 10.1007/s00784-026-06989-3

**Published:** 2026-07-09

**Authors:** Clemens Raabe, Simone F. M. Janner, Michael M. Bornstein, Fabrice A. Dulla, Tobias Zeller, Vivianne Chappuis, Emilio Couso-Queiruga

**Affiliations:** 1 Department of Oral Surgery and Stomatology, School of Dental Medicine, University of Bern, Freiburgstrasse 7, Bern, CH-3010 Switzerland; 2 Clinic of Oral Surgery, University Center for Dental Medicine Basel UZB, University of Basel, Basel, Switzerland; 3 Surgery Center ZIKO Bern, Bern, Switzerland; 4 Department of Oral Health & Medicine, University Center for Dental Medicine Basel UZB, University of Basel, Basel, Switzerland

**Keywords:** Tooth transplantation, Patient reported outcome measures, Patient satisfaction, Treatment outcome, Observer variation, Visual analog scale

## Abstract

**Objectives:**

This study aimed to assess patient-reported outcomes (PROs) and clinician-reported outcomes (CROs) following tooth autotransplantation, and to identify factors influencing PROs and CROs.

**Materials and methods:**

Patients with autotransplanted teeth underwent a follow-up examination and completed visual analogue scale (VAS)-based questionnaires assessing multiple treatment domains. Corresponding items were independently evaluated by three oral surgeons and three general practitioners, based on standardized photographs, periapical radiographs, and digital scans of the region of interest. Inter-rater agreement was assessed, and associations between transplant characteristics and outcomes were analyzed.

**Results:**

The sample comprised 33 patients with 37 autotransplanted teeth and a mean follow-up of 8.5 ± 5.8 years. Patients’ satisfaction exceeded 90% for oral hygiene accessibility and fulfillment of expectations, whereas esthetic satisfaction (81%) and quality-of-life impact (60.5%) were rated lowest. CROs were significantly lower than PROs for esthetic satisfaction, oral hygiene accessibility, and fulfillment of expectations, whereas PROs were lower for quality-of-life impact (*p* ≤ 0.005). Inter-rater agreement among clinicians ranged from poor to fair. Infraposition significantly reduced both PROs and CROs (*p* ≤ 0.05). Additionally, general practitioners assigned significantly lower CROs than to oral surgeons, particularly in the presence of healing sequelae and gingival recession defects (*p* ≤ 0.045).

**Conclusions:**

Tooth autotranslantation was associated with high-long-term patient satisfaction, whereas clinicians rated outcomes more critically. Infraposition was the only variable negatively affecting both PROs and CROs.

**Clinical relevance:**

Despite more critical clinician assessments, patients reported high satisfaction following tooth autotransplantation, supporting this treatment approach as a valuable option for replacement of missing teeth.

## Introduction

In cases of missing or failing teeth, tooth autotransplantation represents a unique, biologically driven and cost-effective treatment modality with promising long-term outcomes in both adolescents and adults [1, 2]. In contrast to prosthetic or implant-based tooth replacement strategies, autotransplantation preserves the regenerative potential of the periodontal ligament, thereby enabling site-specific tissue regeneration and preservation of the alveolar ridge dimensions [3]. These regenerative capacities include the reestablishment of a functional periodontal ligament, preservation and continued development of the alveolar ridge, and corresponding gingival tissues; all physiologic capacities not associated with prosthetic or implant-based tooth replacement strategies [4].

Nonetheless, the success of tooth autotransplantation may be compromised by a range of biological and site-related factors. Sequelae following tooth autotransplantation are frequently observed and may affect both patient and clinician perception of treatment outcomes, potentially resulting in reduced satisfaction [5]. At the biological level, impaired periodontal healing represents the most common complication, often arising from mechanical damage to the donor tooth root and/or prolonged extraoral time during surgery, potentially resulting in root resorption and ankylosis with subsequent infraposition of the transplanted tooth [6]. These conditions may compromise orthodontic alignment, hinder optimal functional integration within the dental arch, and reduce proprioceptive feedback mediated by the periodontal ligament [6]. Pulpal healing may further influence treatment outcomes. While pulp canal obliteration is frequently observed and is considered as a sign of healing, pulpal necrosis requires root canal treatment and may prolong treatment duration. Both scenarios can lead to crown discoloration and/or prolong the overall treatment duration, potentially affecting the patient’s satisfaction [7, 8]. Beyond these biological considerations, the local recipient site conditions may influence both treatment success and patient perception. Horizontal and vertical bone deficiencies with or without soft tissue deficiencies may not fully match the dimensions of the donor tooth, thereby limiting optimal functional and esthetic integration of the transplant [9]. In such scenarios, discrepancies in crown morphology and root anatomy may result in deviations from the ideal tooth form and emergence profile, ultimately leading to suboptimal functional and esthetic outcomes [10].

Given these biological, functional, and esthetic considerations, the overall success of tooth autotransplantation should not be based solely on clinical and radiographic parameters [11]. Patient-reported outcomes (PROs), particularly satisfaction with functional and esthetic results, are increasingly recognized as essential components in evaluating treatment success [12–14]. Although autotransplantation generally yields favorable clinical and radiographic outcomes, esthetic evaluations reveal dissatisfaction rates of up to 18%, and esthetic scores that remain inferior to those of natural dentitions [15, 16]. Other studies have described positive patient perceptions regarding the surgical procedure, postoperative healing period, and overall treatment outcomes [17–19]. Nevertheless, the patient`s perception does not necessarily align with the clinician’s perspective on the functional and esthetic outcomes of tooth replacement strategies, with clinicians typically applying higher and more critical standards than patients [15, 20]. Despite the growing recognition of subjective outcome measures, evidence regarding PROs and CROs following tooth autotransplantation remains limited [21].

Therefore, the primary aim of this study was to assess PROs and clinician-reported outcomes (CROs) of autotransplanted teeth using visual analog scales (VAS). Secondary aims included evaluating the inter-rater agreement among clinicians and identifying patient-, surgery-, and transplant-related factors potentially influencing PROs and CROs.

## Materials and methods

### Study design and ethical approval

This retrospective investigation was conducted at the Department of Oral Surgery and Stomatology, University of Bern, Switzerland, between November 2023 and September 2025. It constitutes Part II of a two-part retrospective cohort study, focusing on subjective outcomes (PROs and CROs), whereas Part I addresses objective findings on treatment survival and success [22]. Ethical approval was obtained from the Ethics Commission of the State of Bern (KEK-BE: 2023 − 01338, Bern, Switzerland). The study adhered to the principles outlined in the Declaration of Helsinki (2013) and complied with the STROBE (Strengthening the Reporting of Observational Studies in Epidemiology) guidelines [23]. Written informed consent was obtained from all participants prior to their enrollment.

Patients who had undergone tooth autotransplantation at the Department of Oral Surgery and Stomatology, University of Bern, Switzerland, between 2000 and 2022 were identified through an electronic search of the institutional clinical database and subsequently contacted by telephone to arrange a single follow-up examination. Patients were eligible if the autotransplanted tooth had been in place for at least 12 months and they consented to a combined clinical and radiographic examination. Patients were excluded if the transplanted tooth had been lost prior to follow-up, if they were pregnant at the time of examination, or if they declined to participate. Details of the surgical protocols for tooth autotransplantation, stratified by root formation stage — immature (incomplete root formation with open apex; Moorrees stages R¼–RC) and mature (apical constriction present; Moorrees stages A½ and Ac) — are reported elsewhere [22, 24].

### Clinical and radiographic examination

The clinical examination included visual inspection of the transplant site for signs of inflammation, assessment of tooth mobility, and evaluation of midfacial gingival recession defects. Bleeding on probing and/or suppuration (BOP/SOP), as well as probing depths (PD), were recorded at six sites per tooth. Occlusal contacts, tooth discoloration, and clinical signs of ankylosis were also assessed. When applicable, restorations were evaluated for structural integrity and functional adequacy. Periapical radiographs of the transplanted teeth were obtained using the paralleling technique with a holder and an intraoral sensor (Xios XG Supreme, Dentsply Sirona Schweiz AG, Baden, Switzerland). Standardized intraoral photographs of the region of interest were obtained from occlusal and lateral perspectives using a digital camera system (Canon EOS 7D, Melville, NY, USA) equipped with a 60 mm macro lens (Canon EF-S 60 mm, Melville, NY, USA) and a macro ring flash (MF-R76, GODOX Photo Equipment Co., Shenzhen, China). Camera settings were standardized (aperture f/25, ISO 200, shutter speed 1/160 s). Full-arch intraoral scans of the maxilla and mandible were also acquired using the TRIOS Move+ system (3Shape, Copenhagen, Denmark).

Autotransplanted teeth were classified as successful based on a combined clinical and radiographic outcome set [22]. Clinical criteria included the absence of inflammatory signs (swelling, sinus tract, or pain), no pain on percussion, the tooth in functional occlusion, probing depths below 3.5 mm, tooth mobility not exceeding grade I, and no need for root canal treatment. Radiographic criteria involved a continuous periodontal ligament space, no evidence of inflammatory or replacement root resorption, no periapical or pararadicular radiolucency, and — in teeth not treated with root canal therapy — pulp canal obliteration or bone ingrowth into the pulp chamber [5, 11, 25].

### Patient-reported outcome measures

Each patient was asked to rate questions addressing past (remembering, Q1-Q5) and present situations regarding incorporation, esthetics, hygiene, overall satisfaction, and willingness to undergo the procedure again on a 100 mm visual analog scale (Q6-Q12, Table 1). Patients with multiple transplants were asked to complete a separate questionnaire for each transplant. All questionnaires were self-completed.

**Table 1:** Items used to assess patient (past and present) and clinician-reported outcomes (present), including the rating for each of the items

Section	Question	Abbreviation	Rating
Retrospective			
Q1	Do you remember which tooth was transplanted?	Tooth identification	Correct tooth / One tooth apart / More than one tooth apart
Q2	Do you recall the surgical procedure?	Surgical recall	Yes / No
Q3	How many days did the pain last following surgery?	Pain duration (healing)	Days
Q4	What was the maximum pain intensity during the healing period?	Peak pain (healing)	VAS (0 = no pain; 100 = worst imaginable pain)
Q5	How frequently did you experience pain on the transplant after healing?	Pain recurrence frequency	VAS (0 = never; 100 = very frequently)
Present			
Q6	I am satisfied with the esthetics of my transplanted tooth.	Esthetic satisfaction	VAS (0 = strongly disagree; 100 = strongly agree)
Q7	I can chew with my transplanted tooth as well as with my other teeth.	Chewing function	VAS (0 = strongly disagree; 100 = strongly agree)
Q8	I can clean my transplanted tooth as well as my other teeth.	Oral hygiene accessibility	VAS (0 = strongly disagree; 100 = strongly agree)
Q9	My transplanted tooth feels like my other teeth.	Tooth perception	VAS (0 = strongly disagree; 100 = strongly agree)
Q10	My transplanted tooth has met my expectations.	Expectation fulfillment	VAS (0 = strongly disagree; 100 = strongly agree)
Q11	If necessary, I would be willing to undergo the procedure again.	Willingness to repeat	VAS (0 = strongly disagree; 100 = strongly agree)
Q12	Has the tooth autotransplantation improved your quality of life?	Quality of life impact	VAS (0 = strongly disagree; 100 = strongly agree)

*Q* Question, *VAS* visual analog scale

### Clinician-reported outcome measures

**Fig. 1:**
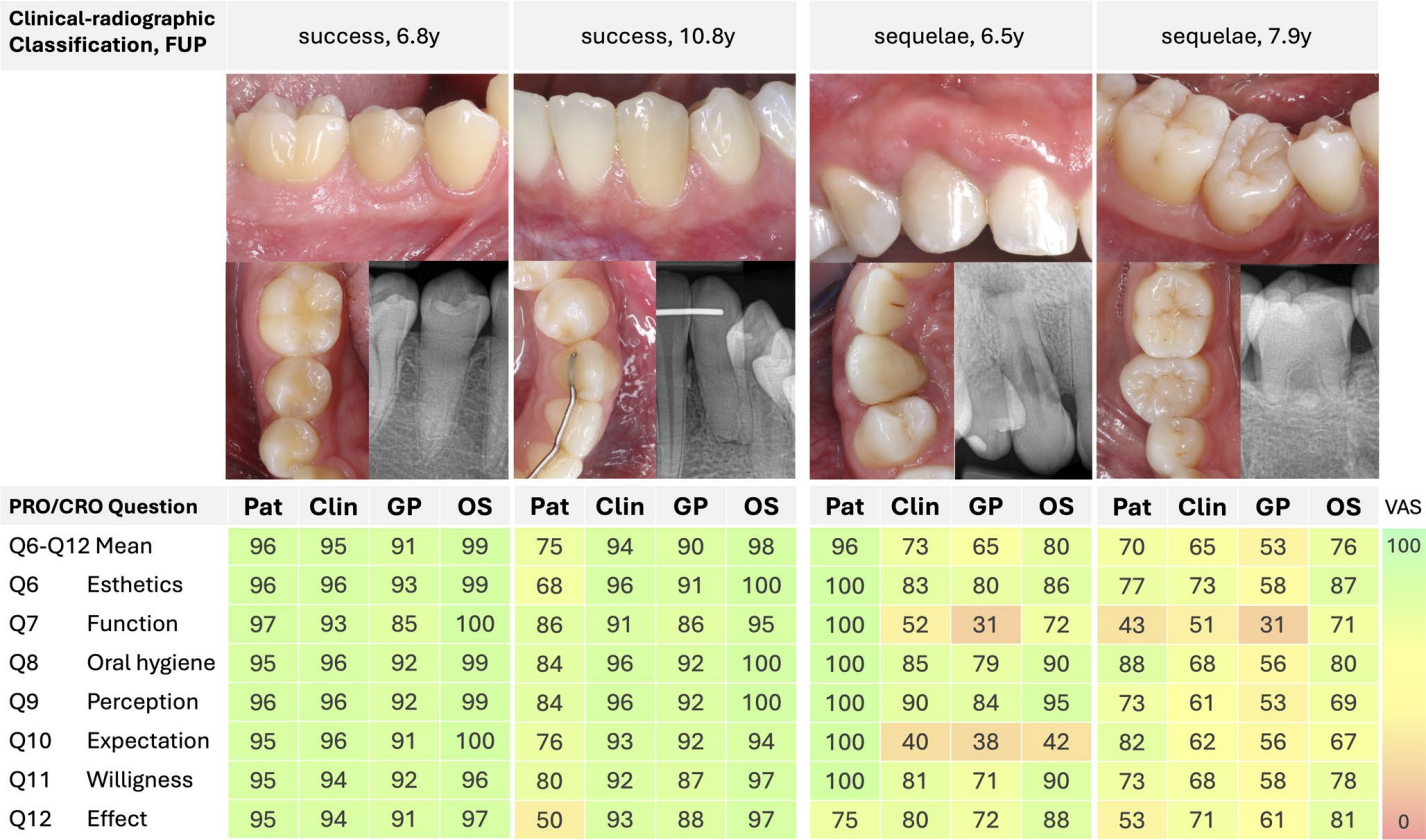
Representative cases of autotransplanted teeth are shown from lateral and occlusal views with periapical radiographs at follow-up (FUP), classified as successful or presenting with sequelae. Visual analogue scale (VAS) scores for patient-reported (PRO) and clinician-reported outcomes (CRO) — including overall clinicians (Clin), general practitioners (GP), and oral surgeons (OS) — are displayed as a color-coded heatmap across questionnaire domains Q6–Q12. Cases illustrate a spectrum ranging from uniformly high PRO and CRO scores (Case 1), through discordant scoring patterns (Cases 2–3), to uniformly moderate-to-low scores (Case 4). Follow-up durations are indicated in years (y). For details on questionnaire items, see Table 1

All available radiographs (historical and current), intraoral photographs, and digital scans were uploaded to an online evaluation platform, together with clinical data on mobility, PD, and BOP/SOP, but without any treatment success classification. Prior to evaluation, all raters participated in a calibration session using reference cases to ensure familiarity with the platform and rating system. Each case was independently assessed by six raters from two professional backgrounds, three board-certified oral surgeons (OS) and three general dental practitioners (GP), none of whom had been involved in the clinical part of the study. Using the same visual analogue scale format as the patient questionnaires, raters scored each case on the clinician-reported counterparts of the PRO domains (Q6–Q12) (Fig. 1).

### Statistical analysis

All analyses were performed with the statistics software R, version 4.4.2 [26]. Baseline characteristics, clinical, as well as PROs and CROs, were descriptively summarized by giving means, medians, and standard deviations for continuous variables and by giving frequencies and percentages for categorical variables. Data were furthermore illustrated with the help of boxplots.

Intraclass correlation coefficients (ICCs) were used to assess rater agreement of CRO scores overall and within clinician groups. Coefficients were further assessed using the well-known grading scale by Cicchetti [27].

Differences between PRO and average CRO scores for Q6-Q12 of both clinician groups and overall clinicians were assessed with the help of non-parametric repeated measures ANOVAs. Note that this robust nonparametric alternative was used because of heteroscedasticity and outliers in the data. In case of significance, post hoc and group-wise repeated measure ANOVAs were performed. P-values were thereby corrected by the method of “Holm”.

Finally, the association of baseline characteristics and treatment outcomes with PROs and average CRO scores of both clinician groups was assessed with the help of robust linear mixed regression models. Parameters were first assessed in an univariable context, and if their p-value was less than or equal to 0.10, they were carried forward into a larger multivariable model to obtain corrected effects and determine possible risk factors/predictors. Goodness-of-fit of models was assessed by looking at residual plots. Multivariable models were checked for collinearity. All p-values lower than or equal to 0.05 were considered statistically significant.

## Results

### Sample characteristics

The study sample comprised 33 patients (26 female, 78.8%; 7 male, 21.2%) with 37 transplanted teeth, who had a mean age at surgery of 16.0 ± 4.6 years and attended a clinical-radiographic follow-up examination after a mean postoperative period of 8.2 ± 5.6 years. Donor teeth were predominantly immature (*n* = 26, 70.3%, versus mature *n* = 11, 29.7%), and consisted of premolars (*n* = 16; 43.2%) and third molars (*n* = 16; 43.2%), followed by canines (*n* = 4; 10.8%) and molars (*n* = 1, 2.7%). Twenty-five donors originated from the maxilla (67.6%) and 12 from the mandible (32.4%). Recipient sites were most frequently in the premolar region (*n* = 27; 73.0%), followed by the incisor/canine and molar regions (*n* = 5 each; 13.5%), with 25 situated in the mandible (67.6%) and 12 in the maxilla (32.4%). In most cases, donor and recipient sites differed (*n* = 32; 86.5%); in five cases (13.5%), a displaced tooth was transplanted to its intended anatomical site.

At follow-up, clinical signs of infection were observed in one patient (2.7%), and pain was reported in another patient (2.7%). The mean maximum probing depth was 3.1 mm (median 3.0; SD 1.0), and mean bleeding on probing was 15.3% (median 16.7; SD 18.6). Tooth mobility was grade 0 in 34 teeth (91.9%) or grade I in 3 teeth (8.1%). Gingival recession defects of ≥ 1 mm were present in 10 cases (27.0%), and infraposition in four (10.8%). Root canal treatment was performed in six transplants (16.2%). No active carious lesions were detected at follow-up; however, restorations were present in 10 teeth (27.0%): root canal access restorations (*n* = 6; 16.2%), interproximal restorations (*n* = 3; 8.1%), and one partial crown (2.7%).

According to the composite outcome set, 24 transplants were classified as successful (64.9%) and 13 (35.1%) exhibited sequelae, including need for root canal treatment (*n* = 6), replacement root resorption with infraposition (*n* = 2), infraposition without signs of root resorption (*n* = 2), apico-marginal lesions (*n* = 1), invasive cervical resorption (*n* = 1), and apical periodontitis (*n* = 1).

### Retrospective patient-reported outcomes

Regarding recall of the transplanted tooth’s final position, correct identification was achieved in 14 of 37 cases (38%). In a further 14 cases (38%), patients pointed to an adjacent tooth instead, and in the remaining 9 cases (24%), they were unable to localise the transplant, having indicated a position more than one tooth away.

Regarding the transplantation procedure, 5 patients (14%) did not recall the surgery or the subsequent healing phase; notably, this subgroup had a follow-up interval ranging from 6.0 to 16.3 years postoperatively. Among the remaining 32 patients who recalled the perioperative period, the mean duration of painful sensations was 4.7 ± 2.9 days, with a mean peak pain level of 34.5 ± 22.0. Following the initial healing phase, patients reported for 15 transplants (40.5%) never experiencing discomfort (VAS 0), while 18 sites (48.6%) showed occasional episodes of discomfort (VAS 11.7 ± 7.8), and four transplants (11.8%) yielded recurrent episodes of discomfort (VAS 70.5 ± 20.8).

### Patient- and clinician-reported outcomes at follow-up

**Fig. 2:**
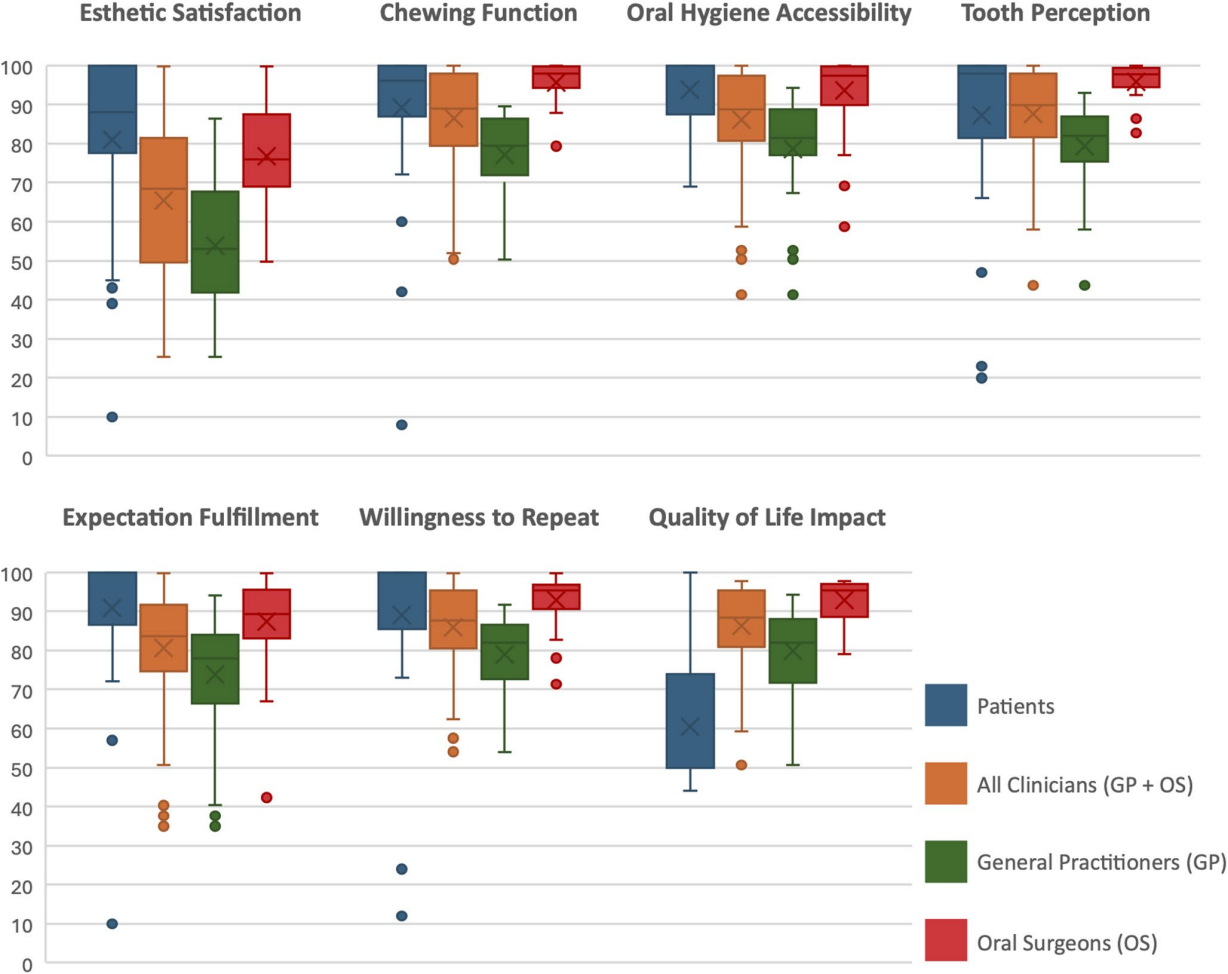
Patient and clinician-reported outcomes for autotransplanted teeth, scored from 0 (complete disagreement) to 100 (complete agreement). Clinician data are shown overall and by specialty (general practitioners (GP), oral surgeons (OS)). Boxplots are presented for each statement, with x denoting the mean

Mean VAS values were significantly higher among patients than clinicians for esthetic satisfaction (81.0 vs. 65.3, *p* < 0.001), oral hygiene accessibility (93.8 vs. 86.0, *p* < 0.001), and expectation fulfillment (90.8 vs. 80.5, *p* = 0.005), with esthetic satisfaction demonstrating the greatest variability. Comparable ratings, within a 5% difference, were observed between patients and clinicians for chewing function (89.3 vs. 86.4), tooth perception (87.2 vs. 87.5), and willingness to repeat the procedure (89.1 vs. 85.9); none of these differences reached statistical significance. Conversely, patients rated the impact on quality of life lower than clinicians (60.5 vs. 86.3, *p* < 0.001). OS consistently assigned higher ratings than GPs, and significant differences were found when comparing ratings across all three groups (patients, GPs, and OS) for all domains (all *p* < 0.001). Further details on the ratings are presented in Table 2; Fig. 2.

**Table 2:** Scores of PROs and CROs regarding the present state of the transplanted tooth (Q6-12). Clinician data are shown overall and by clinical background

Questions	Patients	All clinicians (GP + OS)	GP	OS	*p*-value patients vs. (GP + OS)	*p*-value patients vs. GP vs. OS
Q6, Esthetic satisfaction, VAS	81.0 [88.0] (23.3)	65.3 [68.5] (19.1)	53.9 [53.0] (17.6)	76.7 [76.0] (12.7)	< 0.001***	< 0.001***
Q7, Chewing function, VAS	89.3 [96.0] (19.4)	86.4 [89.0] (13.0)	77.2 [79.3] (11.4)	95.7 [98.0] (5.9)	0.41	< 0.001***
Q8, Oral hygiene, VAS	93.8 [100.0] (8.9)	86.0 [88.8] (14.1)	78.6 [81.3] (14.0)	93.5 [97.3] (9.6)	< 0.001***	< 0.001***
Q9, Tooth perception, VAS	87.2 [98.0] (21.1)	87.5 [89.8] (11.8)	79.3 [82.0] (10.9)	95.8 [97.7] (5.1)	0.91	< 0.001***
Q10, Expectation fulfillment, VAS	90.8 [100.0] (17.7)	80.5 [83.6] (15.4)	73.7 [78.0] (15.8)	87.3 [89.3] (11.8)	0.005**	< 0.001***
Q11, Willingness to repeat, VAS	89.1 [100.0] (20.3)	85.9 [87.7] (11.0)	78.9 [82.0] (10.2)	93.0 [95.3] (6.1)	0.31	< 0.001***
Q12, Quality of life impact, VAS	60.5 [50.0] (17.8)	86.3 [88.4] (10.7)	79.8 [82.0] (10.7)	92.8 [95.3] (5.4)	< 0.001***	< 0.001***
Overall Q6-Q12 mean, VAS	84.5 [88.6] (13.7)	82.6 [85.4] (11.6)	74.5 [76.5] (9.9)	90.7 [90.8] (6.4)	0.39	< 0.001***

*GP* general practitioner, *OS* oral surgeons, *Q* Question, *VAS* visual analog scale

Data presented as Mean [Median] (SD)

*p < 0.05

### Inter-rater agreement in clinician-reported outcomes

Across all examiners, ICCs ranged from 0.23 to 0.39, reflecting poor overall agreement. Within the OS subgroup, ICCs ranged from 0.01 to 0.37, similarly indicating poor agreement. Among GPs, ICCs ranged from 0.29 to 0.48, with poor agreement observed for three questionnaire items and fair agreement for four. Details on the inter-rater agreement are presented in Table 3.

**Table 3:** Inter-rater agreement assessed using ICCs with 95%-CIs for GP (general practitioner) and OS (oral surgeon) groups, and across all examiners. ICC values were interpreted according to Cicchetti (1994) [27]: <0.40 = poor, 0.40–0.59 = fair, 0.60–0.74 = good, and 0.75–1.00 = excellent

Questions	GP	OS	Overall
Q6, Esthetic satisfaction	0.42 (0.22, 0.62)	0.37 (0.17, 0.58)	0.35 (0.21, 0.51)
Q7, Chewing function	0.33 (0.13, 0.54)	0.11 (-0.08, 0.33)	0.23 (0.11, 0.39)
Q8, Oral hygiene	0.41 (0.21, 0.61)	0.29 (0.09, 0.50)	0.38 (0.25, 0.55)
Q9, Tooth perception	0.34 (0.14, 0.55)	0.04 (-0.13, 0.26)	0.24 (0.12, 0.40)
Q10, Expectation fulfillment	0.48 (0.29, 0.67)	0.22 (0.03, 0.44)	0.39 (0.25, 0.55)
Q11, Willingness to repeat	0.29 (0.09, 0.51)	0.06 (-0.12, 0.28)	0.26 (0.14, 0.42)
Q12, Quality of life impact	0.45 (0.25, 0.64)	0.01 (-0.15, 0.23)	0.30 (0.17, 0.46)

* Q* Question

### Factors associated with patient- and clinician-reported outcomes

In the multivariable analysis, PROs and CROs were significantly lower if the transplant showed infraposition (PROs − 8.12, CROs − 14.20; *p* ≤ 0.05). Additionally, CROs were significantly lower for GPs compared to OS (-15.03, *p* < 0.001), in transplants with sequelae (-4.50, *p* < 0.001), and in the presence of gingival recessions (-4.21, *p* = 0.045). Other patient-, surgical-, or transplant-related characteristics, such as patient age or sex, mismatch of donor and recipient site, PD, BOP, were not associated with PROs or CROs in the multivariate analyses. Further details are presented in Tables 4 and 5.

**Table 4:** Univariable screening of PRO and CRO scores. Items with *p* ≤ 0.10 were carried forward to the multivariable model to identify potential risk factors/predictors

Characteristic	Patient-reported outcomes	*p*-value	Clinician-reported outcomes	*p*-value
Gender		0.70		0.78
Female	Baseline		Baseline	
Male	1.39 (-5.60, 8.38)		-0.89 (-7.16, 5.38)	
Age at Surgery	-0.13 (-0.74, 0.48)	0.67	-0.32 (-0.86, 0.22)	0.24
Time until follow-up -5y	0.17 (-0.32, 0.67)	0.49	0.47 (0.06, 0.88)	0.03*
Position donor tooth		0.89		0.40
Incisors/Canines	Baseline		Baseline	
Premolars/Molars	-1.36 (-7.57, 4.85)		-3.26 (-11.02, 4.51)	
Jaw donor tooth		0.67		0.17
Mandibula	Baseline		Baseline	
Maxilla	-1.36 (-7.57, 4.85)		-3.73 (-9.18, 1.72)	
Position recipient tooth		0.89		0.40
Incisors/Canines	Baseline		Baseline	
Premolars/Molars	0.59 (-8.12, 9.29)		-3.26 (-11.02, 4.51)	
Jaw recipient tooth		0.03*		0.21
Mandibula	Baseline		Baseline	
Maxilla	6.40 (0.66, 12.1)		3.39 (-1.99, 8.77)	
Donor and recipient tooth same?		0.89		0.37
No	Baseline		Baseline	
Yes	-0.56 (-8.50, 7.39)		3.20 (-3.98, 10.4)	
Maximum probing depth	-0.22 (-2.58, 2.14)	0.86	-0.64 (-2.76, 1.48)	0.55
Mean bleeding on probing	0.03 (-0.09, 0.15)	0.64	-0.01 (-0.13, 0.10)	0.81
Gingival recession defects		0.18		0.07
No	Baseline		Baseline	
Yes	-3.96 (-9.83, 1.91)		-4.59 (-9.61, 0.44)	
Classification		0.37		< 0.001***
Success	Baseline		Baseline	
Sequelae	-2.24 (-7.21, 2.72)		-7.72 (-11.6, -3.82)	
Restoration		0.86		0.36
None	Baseline		Baseline	
Present	-0.46 (-5.63, 4.72)		-2.17 (-6.86, 2.53)	
Infraposition		0.048*		< 0.001***
No	Baseline		Baseline	
Yes	-9.05 (-18.0, -0.06)		-16.29 (-23.56, -9.03)	
Apicoectomy		0.15		0.92
No	Baseline		Baseline	
Yes	-3.98 (-9.48, 1.52)		-0.25 (-5.60, 5.10)	

*p < 0.05

**Table 5:** Items selected for inclusion in the multivariable models for patient- and clinician-reported outcomes, and both. N/A: item did not meet the univariate inclusion threshold (*p* > 0.1) and was not carried forward

Characteristic	Patient-reported outcomes	*p*-value	Clinician-reported outcomes	*p*-value
Intercept	90.89		96.15	
Infraposition		0.05*		< 0.001***
No	Baseline		Baseline	
Yes	-8.12 (-16.32, 0.08)		-14.20 (-20.9, 7.53)	
Group		N/A		< 0.001***
OS	N/A		Baseline	
GP	N/A		-15.03 (-16.2, -13.9)	
Recession		N/A		0.045*
No	N/A		Baseline	
Yes	N/A		-4.21 (-8.32, -0.09)	
Classification		N/A		< 0.001***
Success	N/A		Baseline	
Sequelae	N/A		-4.50 (-7.67, -1.33)	
Time until follow-up -5y	N/A	N/A	0.20 (-0.12, 0.52)	0.21
Jaw recipient tooth		0.07		N/A
Mandibula	Baseline		N/A	
Maxilla	4.87 (-0.33, 10.08)		N/A	

*p < 0.05

## Discussion

This study, conducted as part II of a retrospective cohort study, evaluated patient- and clinician-reported outcomes following tooth autotransplantation, alongside the impact of patient-, surgical-, and transplant-related characteristics on these outcomes. Overall, patients reported high levels of satisfaction across multiple domains. In contrast, clinician rated outcomes significantly lower with respect to esthetic satisfaction, oral hygiene accessibility, and expectation fulfillment. Interestingly, clinicians perceived the impact on quality of life more favorably than patients. Infraposition negatively affected both PROs and CROs, whereas lower CROs were additionally associated with assessment by GPs, as well as with the presence of healing sequelae or gingival recession defects.

Most patients recalled having undergone the intervention and, despite the invasive nature of the procedure involving one or multiple surgical sites, reported only moderate pain levels during the initial healing phase, supporting the overall clinical acceptability of tooth autotransplantation. These findings are consistent with other studies, in which the majority of patients recalled the surgery and reported a mean pain level of 3.1 ± 2.4 on a scale of 10, closely approximating the 34.5 ± 22.0 on a scale of 100 observed in the present study [18, 28]. Following the initial healing phase, only a minority of patients reported recurrent episodes of discomfort at the transplanted tooth, and only one patient experienced discomfort at the time of follow-up. Notably, this tooth was transplanted following complete root formation, had undergone root canal treatment, and presented with increased probing depths, suggesting compromised periodontal conditions as a possible contributing factor.

Only approximately one-third of patients were able to correctly identify the transplanted tooth within their oral dentitions, while another third misidentified it by one tooth, and the remaining third deviated further. This finding contrasts with previous reports describing a high level of certainty regarding the identity of their transplanted tooth, although patients in those studies were not asked to clinically identify the tooth intraorally [18, 28]. Additionally, the high scores recorded for perceived “tooth-like” sensation suggest that autotransplanted teeth may become functionally and perceptually integrated into the natural dentition to a degree at which patients can no longer clearly distinguish them from adjacent teeth. This observation is likely related to the preservation of the periodontal ligament and its associated proprioceptive capacity. The long mean follow-up of 8.5 ± 5.8 years may further contribute to this observation, as prolonged functional loading and tissue remodeling may facilitate biologic and functional integration of the transplant. Interestingly, similarly high levels of satisfaction regarding tooth-like perception have been reported for dental implants despite the absence of a periodontal ligament [29].

Patients reported mean satisfaction levels exceeding 90% for oral hygiene accessibility and expectation fulfillment, closely followed by chewing function, and willingness to repeat the procedure. Previous studies have similarly reported high levels of tooth-like perception and oral health maintenance, though they did not assess the broader range of PROs domains evaluated here [18, 28]. In contrast, esthetic satisfaction and perceived improvement in quality of life were rated lower. These are consistent studies investigating premolar autotransplantation to the anterior maxilla, reporting that 71–73% of patients were satisfied with the esthetic outcome, 9–18% were fairly satisfied, and 11–18% were dissatisfied [8, 15]. Compared with natural dentitions not requiring tooth replacement therapy, esthetic outcomes following autotransplantation for lateral incisor replacement were reported to be significantly inferior [16]. Interestingly, the moderate impact on quality of life reported by the patients was also found in another study, which evaluated the OHIP-14 and found values of 1.2 ± 0.21 [18]. This limited effect is most likely attributable to the limited sensitivity of the single-item quality-of-life question used in the present study, as well as the known limited responsiveness of the OHIP-14 for single missing teeth in non-esthetic sites [30]. Interestingly, when comparing the patient satisfaction across tooth replacement therapies, both autotransplantation and dental implants appear to achieve similarly high patient satisfaction scores (9 + out of 10), despite fundamentally different biological and clinical characteristics [31].

While previous studies consistently report favorable patient perceptions, the range of PRO domains assessed remains limited. To the best of the authors’ knowledge, none have simultaneously evaluated CROSs using corresponding questionnaire items, as performed in the present investigation. In contrast to the generally favorable PROs, clinicians were significantly less satisfied with regard to esthetic satisfaction, oral hygiene accessibility, and expectation fulfillment. Contrarily, the perceived effect on quality of life was rated higher by clinicians compared to patients, reflecting a greater expectation of treatment benefit from the professional perspective. This aligns with the literature, consistently demonstrating that the clinical outcomes considered most important by clinicians, such as survival, resorption, and pulp vitality, do not necessarily align with the patient-prioritized outcomes, including satisfaction, quality of life, and function [11]. The potential sources of esthetic dissatisfaction following autotransplantation have been characterized previously, identifying insufficient color match of the crown, excessive gingival width around the transplant, and unfavorable gingival contour around the transplant [15]. Indeed, evidence suggests no significant correlation between professional and patient assessments of esthetic results [20, 31, 32]. Interestingly, significant differences in CROs were observed between OS and GP, with GPs reporting lower satisfaction across all domains. This observation is consistent with studies comparing ratings by investigators of different specialties or experience levels [33]. The more favorable rating observed among OS may reflect their familiarity with the range of biologic and esthetic outcomes following autotransplantation, whereas the expectations of GPs may be oriented toward the gold standard of untreated natural teeth. These findings highlight the importance of appropriate expectation management and raising awareness regarding the inherent variability of outcomes following tooth autotransplantation, a highly technique-sensitive procedure whose esthetic results depend strongly on the morphological and dimensional compatibility between donor tooth and recipient site. Moreover, unmet esthetic expectations may prompt restorative or prosthodontic workflows, potentially involving crown preparation. Such interventions should be approached with caution, as they may damage the pulp replacement tissue, which differs in composition from regular pulpal tissue and may be more susceptible to necrosis and infection [34, 35].

This study has several limitations that should be acknowledged. First, the retrospective design and relatively small sample size in a university-based setting limit the generalizability of the findings. Second, the assessment of PROs and CROs at a single follow-up visit introduces the potential for recall bias. The substantial variability in follow-up time may further confound comparisons across patients. Third, the use of non-validated, study-specific VAS-based questionnaires limits the comparability of results with other studies and may not fully capture the multidimensional nature of patient satisfaction and quality of life. Finally, clinician ratings were based on standardized photographs, radiographs, and digital scans rather than direct clinical examination, which may have restricted the information available for CRO assessment, particularly regarding functional parameters such as occlusion and tooth mobility. Future studies should employ prospective, multicenter designs with larger cohorts and validated, autotransplantation-specific PROMs to improve generalizability and comparability. Longitudinal assessments would clarify how satisfaction evolves over time and in response to secondary interventions.

## Conclusion

Tooth autotransplantation was generally associated with high patient satisfaction, with most patients unable to distinguish the transplanted tooth from their natural dentition. In contrast, CROs were significantly lower than PROs regarding esthetic satisfaction, oral hygiene accessibility, and expectation fulfillment, whereas PROs were lower for quality-of-life impact. Infraposition was the only factor negatively associated with both PROs and CROs, while healing sequelae, gingival recession defects, and clinician background further influenced CROs.

## Data Availability

The data that support the findings of this study are available from the corresponding author upon reasonable request.

## Declarations

### Generative AI and AI-assisted technologies in the writing process

During the preparation of this work, the authors used ChatGPT/OpenAI for language editing purposes. After using this tool, the authors reviewed and edited the content as needed and took full responsibility for the content of the publication.
